# The 24th annual Nucleic Acids Research Web Server Issue (2026)—and a novel concept for the future

**DOI:** 10.1093/nar/gkag669

**Published:** 2026-07-06

**Authors:** Dominik Seelow

**Affiliations:** Exploratory Diagnostic Sciences, Berlin Institute of Health at Charité – Universitätsmedizin Berlin, 10117 Berlin, Germany

This is the 24th special issue for Web Servers. It is rather “thinner” than the last, featuring 47 web servers, for likely reasons that I will discuss later in this editorial.

One web server that deserves particular mention is https://bio.tools, a collection of applications for life sciences. While not a classical scientific web server, websites like bio.tools are urgently needed because they provide a useful, albeit far from complete, overview of the many tools and databases that have emerged over the last few years or even decades. Adding new resources is straightforward—I noticed that some of my own applications were not listed and found it simple to add new resources to the site. I would like to take this opportunity to encourage you to register your own applications. I think that this resource will greatly enhance their findability.

My second plea goes not to the developers but to the users of computational resources: Please cite the databases and applications you use! Most developers of web servers will be aware of a marked disparity between the actual usage of their tools and the number of citations they receive. Few would forget to cite the wet-lab methods they use or to name the cDNA clones they work with, yet computational resources are too often treated differently. For small groups in particular, proper citation is essential to securing funding for further development, or even maintenance. Running a web server or a large database is a costly undertaking. I am a strong advocate of free software, but this cannot be sustained without proper credit being given. Moreover, reproducibility requires a precise description of methods, which of course includes dry-lab tools.

An ongoing trend across all areas is the use of large-language model-based interfaces, i.e. chatbots. Whenever a proposal includes a chatbot, I spend some time talking to it, but the results are often disappointing. While entering questions may be easier than using a classical web interface, the answers must be accurate and reflect the knowledge available at the time of the query. If LLMs are used in a scientific project, they should provide complete and up-to-date information, especially when they cover quickly advancing topics. LLMs connected to external data sources and querying these in real-time (e.g. via the Model Context Protocol) might offer a path forward. However, so far, I would still rather visit an official website about contraindications of a drug than rely on an LLM, which may not have access to the latest studies.

This year’s Web Server Issue includes many applications from various areas that may support your research. A complete list is given in Table 1 below. As always, I tried to group the manuscripts into classes. This is, however, difficult because many clearly can fall into several categories. I would encourage readers not to confine their attention to web servers in their own field, but to read the full list and visit any application that might prove useful. I do this myself whenever a new NAR Database Issue is published and often discover resources I was unaware of.

**Table 1.** List of the web servers included in the 2026 NAR Web Server Issue

**General**		
bio.tools	Web service to find life-science applications	https://bio.tools/
**Cancer/medicine/cheminformatics/synthetic biology**		
CEDAR	Resource for cancer epitopes	https://cedar.iedb.org/
DeepCYP	Prediction of CYP450 enzymes-mediated metabolism	https://deepcyp.scbdd.com/
DNAviWEB	Gel-based sequencing of liquid biopsies	https://dnavi.sc.hpi.de/
Ligify	Prediction of small molecule biosensors	https://ligify.groov.bio/
PLATE-VS	Protein–Ligand Affinity-based Target Evaluation	https://www.drugbench.org/
SynCraft	ADMET-aware retrosynthesis	https://syncraft.denglab.org/
TridentSynth	Retrobiosynthesis to synthesize small-molecules	https://tridentsynth.lbl.gov/
**Genomics**		
ChIP-Atlas	Exploration of ChIP-seq, ATAC-seq, and Bisulfite-seq data	https://chip-atlas.org/
ChromoMapperWeb	Evaluation of genome alignments	https://chromomapperweb.ceinge.unina.it/
FABIAN-variant	Effects of DNA variants on transcription factor binding	https://www.genecascade.org/fabian
Galaxy	The Galaxy platform 2026	https://galaxyproject.org/
RegRegSEA	Enrichment analysis of epigenomic data	https://web.ccb.uni-saarland.de/regregsea/
SNPnexus	Web-based variant annotation	https://snpnexus.org/
T2T-Hub	Platform for telomere-to-telomere genomes	https://bis.zju.edu.cn/t2thub
**Lab work**		
PrimerWeaver	Primer design	https://ignea.lab.mcgill.ca/primerweaver
SNIPSNP	Designing homology directed repair reagents for CRISPR/Cas9	https://crisprtools.org/snipsnp
**Microbiology/virology/phylogeny**		
HERA	Visualization of plasmids and other circular DNA molecules	https://web.ccb.uni-saarland.de/hera/
Microbe Decoder	Uncover what microbes are doing in your system	https://www.microbe-decoder.org/
PhaBOX	Viral contigs in metagenomic data	https://phage.ee.cityu.edu.hk/
**Nucleic acids**		
ARTEM	Analysis of nucleic acid 3D structures	https://artemserver.genesilico.pl/
GEAR Genomics	A collection of applications for molecular biology and genomics	https://www.gear-genomics.com/
RNAanalyzer3	Tools for RNA analysis	https://rnaanalyzer.bioapps.biozentrum.uni-wuerzburg.de/
ShapeRNA	Analysis of RNA secondary structure and function	https://shaperna.com/
**Proteins**		
AlphaFind	Similarity search in AlphaFold DB	https://alphafind.ics.muni.cz/
Atomic Charge Calculator	Calculation of partial atomic charges	https://acc.biodata.ceitec.cz/
BilboMD	SAXS and AlphaFold-guided modeling	https://bilbomd.bl1231.als.lbl.gov/
CATVariant	Protein Variant Analysis	https://catvariant.khoa.ngo/
CB-Dock	Protein–ligand (blind) docking	https://cadd.labshare.cn/cb-dock3/
DeepKinomeWeb	Kinase inhibitor screening	https://str.kribb.re.kr/deepkinome
EnzymeMiner	Automated enzyme discovery	https://loschmidt.chemi.muni.cz/enzymeminer/
FoldDelay	Explore delays in co-translational protein folding	https://folddelay.switchlab.org/
HMMER	Homology search	https://www.ebi.ac.uk/Tools/hmmer
i-gRINN	Residue interaction energy calculation	https://grinn.bio-cloud.site/
PEP-EDIT	Rapid editing and generation of peptide representations	https://pep-edit.rpbs.univ-paris-diderot.fr/
PockFlex	Protein pockets	https://pockflex.rpbs.univ-paris-diderot.fr/
PROPTIMUS LIVE	α-Carbon optimization of proteins	https://proptimus.ceitec.cz/live
PythiaStudio	Deep learning-based protein engineering	https://pythiastudio.wulab.xyz/
SGGly	N-linked glycosylation prediction	https://biosig.lab.uq.edu.au/sggly/
SPICE	Structure of protein complexes	https://spice.cs.ucr.edu/
SPSignal	Nuclear localization and export signals	https://sps.cragenomica.es/
xBind	Cross-molecular protein binding-site prediction	https://fusion.cs.vt.edu/xBind
**Systems biology**		
ChEA-KG	Transcription factor enrichment analysis and network visualization	https://chea-kg.maayanlab.cloud/
COMMBAT	Expression of biosynthetic gene clusters	https://www.commbat.uliege.be/
DAVID	Functional annotation and enrichment analysis of genes	https://davidbioinformatics.nih.gov/
LiMeNEx	Lipid Metabolism Network Explorer	https://limenex.raylab.iiitd.edu.in/
ProteinNetworkSight	Transforming co-expression patterns into therapeutic insights	https://proteinnetworksight.jce.ac/

This year, I was happy to be supported by a member of our Early Career Investigator Advisory Board: Yuxuan Du from the University of Texas at San Antonio helped me with two manuscripts and will continue to be involved in the Web Server Issue. I also want to thank my fellow editors Janusz M. Bujnicki (International Institute of Molecular and Cell Biology, Warsaw) and Dan Rigden (University of Liverpool) who took care of the proposals and manuscripts that may have raised a conflict of interest for me.

**Our technical review team was a bit smaller this year and consisted of:**

- Andrea Cappannini *Université libre de Bruxelles (ULB), Brussels, Belgium*
- Jan-Paul Lerch *Charité-Universitätsmedizin Berlin, Berlin, Germany*
- Sunandan Mukherjee *Międzynarodowy Instytut Biologii Molekularnej i Komórkowej w Warszawie, Warsaw, Poland*
- Shusruto Rishik *Universität des Saarlandes, Saarbrücken, Germany*
- Georges Pierre Schmartz *Administration des contributions directes, Luxembourg*
- Adam Simpkin *University of Liverpool, Liverpool, UK*
- Dominik Sordyl *Międzynarodowy Instytut Biologii Molekularnej i Komórkowej w Warszawie, Warsaw, Poland*

Thank you all, you did an amazing job, as every year. Without you, and of course without the expertise of the numerous anonymous referees, this Issue would not have been possible. The same applies to the whole team at NAR, especially Meera Solanki, who managed to organize me to keep the Issue’s tight deadlines.

## The future will bring changes

I normally end my editorial with acknowledgements. However, this year, there is more to report. As I wrote earlier, this year’s Issue includes fewer manuscripts than usual. While the number of proposals remained broadly stable in recent years, the number of accepted manuscripts has steadily declined. Two major reasons are our policy to require free commercial use and our tight deadlines, both for submitting a proposal and for final acceptance of a manuscript, with the latter impeding larger revisions.

When the Web Server Issue was introduced in 2003, web servers were far less complex than they are today. On the other hand, setting up a working web application was much more difficult: CSS support was buggy and Mozilla and Internet Explorer needed different JavaScript code. Much has changed since then. Numerous frameworks now make the creation of web applications quite straightforward, at least on the technical side, and vibe coding makes developing a working web server easier still.

I, and my fellow editors, believe that it is time to adapt the Web Server Issue to these changes. In the past, a large part of the selection process for new proposals involved checking whether a web application was simple to use, well-documented, freely accessible, and technically sound. We required the underlying methods to be validated in a separate, peer-reviewed publication. The internal review conducted before authors were invited to submit a manuscript was therefore focussed on identifying technical issues, rather than on assessing scientific quality, which requires the expertise of referees from the field addressed by the web server. Considerable time was spent on this technical review at the expense of time for major revisions. The relatively short time between submission of a proposal and our internal deadline for acceptance made revisions beyond fixing bugs or adding small features more or less impossible: it is of course rarely feasible to rewrite an application or to perform wet-lab validation within two or three weeks. This frequently left a difficult choice: to accept an imperfect manuscript in the hope that the authors would act on the referees’ advice, or to reject one that might have been salvaged with additional time.

One conclusion is that modern web applications simply need more time to be carefully evaluated. We have therefore decided to remove our deadlines. From November, proposals can be submitted throughout the year, allowing much more time for revisions. All manuscripts accepted by approximately 1st May of a given year will be compiled into that year’s special issue. However, they will be available online and indexed in PubMed as soon as they are accepted.

This approach has another major benefit: with more time, we will no longer require the underlying methods to have been published elsewhere anymore. Prior publication remains allowed, provided it does not describe the web server itself, and it may substantially reduce review times and revision cycles if the method has already been validated. However, the choice will be entirely up to you as authors.

But this is not all. The issue previously included a section for stand-alone software. As referees will now have more time to install and test such tools, we will once again accept them. We will however require containerized versions to simplify testing and eventually deployment whenever possible.

A final significant change is that we will allow restriction to non-commercial use. I remain a huge fan of open access, but many datasets or tools that authors may wish to include in applications often come with more restrictive licences. We are also aware that funding is dwindling which may create hardware capacity problems and it seems unfair that companies able to pay for a service compete against academic groups that cannot.

While this is largely advantageous, it carries one drawback: In the past, I have tried to communicate reasonably detailed reasons for rejecting proposals, particularly when I could see ways to improve an application for potential resubmission. The new model is likely to attract considerably >250–300 proposals we have received annually in recent years, and I will often not have time to explain a rejection in detail. However, this is normal for presubmission enquiries and a more even distribution of proposals over the year should drastically reduce the time to an initial decision (invitation to submit or rejection).

**In summary:**

- Web applications may be based on unpublished methods.
- We allow restriction to non-commercial use.
- From November you can submit proposals throughout the year.
- The Web Server Issue will still appear on 1 July, but the manuscripts will be published and listed in PubMed after acceptance.

You will find an updated guide to authors and an overview of the changes at https://academic.oup.com/nar/pages/Submission_Webserver.

